# The appearance of joint manifestations in the Swiss inflammatory bowel disease cohort

**DOI:** 10.1371/journal.pone.0211554

**Published:** 2019-04-30

**Authors:** Aimee Hiller, Luc Biedermann, Nicolas Fournier, Matthias Butter, Stephan R. Vavricka, Adrian Ciurea, Gerhard Rogler, Michael Scharl, for the Swiss IBD Cohort Study Group

**Affiliations:** 1 Department of Gastroenterology and Hepatology, University Hospital Zurich, University of Zurich, Zurich, Switzerland; 2 Institute of Social and Preventive Medicine (IUMSP), Lausanne University Hospital, Lausanne, Switzerland; 3 Department of Rheumatology, University Hospital Zurich, Zurich, Switzerland; 4 Zurich Center for Integrative Human Physiology, University of Zurich, Zurich, Switzerland; Holbæk Hospital, DENMARK

## Abstract

**Background/Aims:**

Extraintestinal manifestations (EIM) involving joints, skin, eyes and liver represent an important problem in the treatment of IBD patients. The aim of this study was to identify factors that are associated with the occurrence of joint EIM and therefore allow an early diagnosis and guide medical treatment.

**Methods:**

We studied clinical and epidemiological data from 3298 patients included in the Swiss IBD Cohort Study (SIBDCS), 1860 suffered from Crohn’s disease (CD) and 1438 from ulcerative colitis or IBD unclassified (UC/IBDU).

**Results:**

We found female gender as well as a longer disease duration and activity (specified as CDAI or MTWAI, respectively) to be related to the appearance of arthritis/arthralgia, but also sacroiliitis/ankylosing spondylitis in IBD patients. IBD patients with arthritis/arthralgia or sacroiliitis/ankylosing spondylitis were more often treated with anti-TNF and patients with arthritis/arthralgia underwent more often IBD-related surgeries. We revealed that eye or skin EIM were more frequent in patients with arthritis/arthralgia or sacroiliitis/ankylosing spondylitis. In multivariate analysis, we confirmed female gender, longer disease duration, IBD-related surgery, presence of other EIM and treatment with anti-TNF to be independent risk factors for the onset of arthritis/arthralgia in CD and UC/IBDU patients.

**Conclusion:**

In this study, we demonstrated that markers for a more severe disease course were associated with the onset of joint EIM in IBD patients. Our data suggest that in particular females under anti-TNF treatment and patients suffering from non-joint and/or IBD-related surgery should be close and carefully monitored for presence of arthritis or sacroiliitis/ankylosing spondylitis.

## Introduction

Up to 47% of all inflammatory bowel disease (IBD) patients suffer from extraintestinal manifestations (EIM) [1–3]. Musculoskelettal manifestations in general are the most common EIM in IBD patients with peripheral arthritis being the most frequent subtype [2, 3]. It is obvious that patients with a high-risk profile for such EIM should be closely monitored (as well as treated early). Other organs affected by the intestinal disease are skin, bile duct and eyes [3]. EIM may occur before the actual intestinal disease is diagnosed [1–3]. Furthermore, presence of EIM plays an important role in a patient’s quality of life [4–6].

IBD-associated musculoskelettal EIMs belong to the group of spondyloarthritis (SpA). Depending on their main symptoms, SpA can be divided into axial and peripheral SpA which both can occur in IBD patients either independently or in combination [7]. Peripheral SpA encompasses a number of different musculoskeletal entities: arthralgia, peripheral arthritis, enthesitis and dactylitis. In general, IBD-associated peripheral arthritis is a non-erosive, inflammatory arthropathy and can in many cases be grouped into two subtypes according to Orchard et al. [8] Patients suffering from type 1 exhibit joint pain as well as swelling or effusion that affects less than five joints, most frequently the large joints of the lower limb. Symptoms are often acute, self-limiting, without permanent joint damage and correlate with acute IBD flares. Patients suffering from type 2 exhibit joint pain that affects more than five joints with a symmetric distribution that is predominantly affecting the upper limbs. Symptoms are independent of luminal IBD inflammation and often continue for months or years. Prognosis of peripheral arthritis is mainly a good one and chronic or erosive complications are rare [9, 10]. The main feature of IBD-related axial spondyloarthritis (axSpA) is the involvement of the axial skeleton (sacroiliitis and spondylitis). AxSpA can be divided in a non-radiographic and a radiographic form, depending on whether conclusive evidence for sacroiliacal joint damage can be seen on conventional radiographs. The radiographic form corresponds to ankylosing spondylitis (AS). Prognosis of axial disease is highly variable and linked not only to inflammation, but also to new bone formation, potentially leading to ankyloses. The treatment of axial and peripheral SpA in IBD patients includes non-pharmacological and pharmacological treatments. While non-steroidal anti-inflammatory drugs can be used for all manifestations, conventional disease modifying anti-rheumatic drugs (sulfasalazine, methotrexate, leflunomide) are only effective in the presence of peripheral arthritis (objective synovitis) and not effective in other peripheral joint manifestations like arthralgia, enthesitis and dactylitis or in axial disease. Depending on disease activity, TNF inhibitors may be indicated in refractory cases [11]. Of note, in IBD, only 10% of all patients with joint complaints finally fullfil the SpA criteria [12].

Crohn’s disease (CD), female gender and higher age of the patients were found to be predisposing factors for developing joint EIM in IBD patients [13] and recent studies also suggest a genetic background for developing joint EIM [14]. The so-called gut-joint axis hypothesizes that activated lymphocytes from the gut migrate to the joints and initiate the onset of EIM in the joints [5, 15, 16]. To optimize patient care, it is important to identify predisposing factors for the occurrence of EIM in IBD patients in order to initiate an appropriate and early treatment of both the actual disease and the EIM. This might help to prevent a more severe disease course. For this purpose, we analyzed the patient data of the well-characterized patient collective of the SIBDCS to identify correlation between the presence of joint EIM and epidemiological and clinical risk factors.

## Patients and methods

### Patient data

Data were collected from the Swiss nationwide Swiss IBD Cohort Study (SIBDCS) patient database. The SIBDCS represents a multicenter, prospective observational, population-based study that is funded by the Swiss National Science Foundation (SNSF). The SIBDCS was implemented in all regions of Switzerland in 2006 by a multidisciplinary effort by gastroenterologists, pathologists, psychologists and bioinformatics specialists. For inclusion in the SIBDCS, IBD patients must demonstrate an IBD diagnosis established at least 4 months prior to study entry. Patient data are prospectively collected via specific questionnaires once a year and submitted to a central database. Questionnaires are exhibited in the Supplemental material section of the manuscript. Inclusion and exclusion criteria have been described in detail previously [17]. In our study, we included 3298 IBD-patients in total. 1860 were CD and 1438 were UC/IBD unidentified (IBDU) patients. Data on musculoskeletal conditions thought to be related to IBD were collected through a questionnaire completed at enrollment and follow-up visits by either a gastroenterologist, a study nurse or a research assistant. The presence of arthralgia/peripheral arthritis could be indicated either as yes or still ongoing, as was the presence of sacroiliitis/ankylosing spondylitis. No specific training for evaluation of musculoskeletal conditions was provided to the assessors. We have therefore chosen to only look at peripheral joint manifestations.

### Study design

First, the total patient collective of 3298 patients was divided into two groups: patients with arthritis/arthralgia *vs*. patients without any signs arthritis/arthralgia. In a second step, we analyzed the total patient collective of 3298 patients according to the presence or absence of sacroiliitis/ankylosing spondylitis.

We then analyzed the association of arthritis/arthralgia or sacroiliitis/ankylosing spondylitis, respectively and the following factors: 1) Epidemiological factors such as gender, age at diagnosis, age at latest follow-up, disease duration, family history, smoking status (at diagnosis and latest follow-up) and BMI. 2) Disease characteristics such as initial disease location, last disease location, disease activity (Modified Truelove and Witts activity index, [MTWAI] and CD (Crohn’s disease activity index, [CDAI]) and complications as stenosis, fistula, abscess, anemia and Vitamin B12 levels (at latest follow-up) as well as other EIM and IBD-related surgery. 3) Medical treatment including the following medications: 5-ASA, antibiotics, steroids, immunomodulators (azathioprine, 6-mercaptopurin), anti-TNF, methotrexate, calcineurin inhibitors. 4) Longitudinal data on joint EIM. Non-joint EIM included erythema nodosum, pyoderma gangrenosum, oral and aphtous ulcers, uveitis, iritis and PSC. Malabsorption syndrome with consequent anemia and vitamin B12 deficiency was also considered as a complication. To assess disease activity for UC (Modified Truelove and Witts activity index, MTWAI) and CD (Crohn’s disease activity index, CDAI), disease activity measures were normalized to a value between 0 and 100 and expressed as an activity index. IBD medication was documented by the treating physician in the specific SIBDC questionnaires at patient inclusion and follow-up visits as well as by the patient questionnaires.

### Statistical analysis

All statistical analyses were carried out using the Stata Software (v. 14.2, StataCorp, College Station, TX, USA) and the R software (v. 3.3.1, The R Foundation for Statistical Computing, Vienna, Austria). Normal QQ-plots were used to assess distribution of continuous data. Gaussian-distributed data were reported as mean, standard deviation and range, while non-Gaussian data were presented as median, interquartile range and range. Differences in means between two independent groups for Gaussian-distributed data were assessed using the Student’s t-test. Differences in distribution locations between two independent groups for non-Gaussian data were assessed using the Mann-Whitney-Wilcoxon rank-sum test. Categorical data were presented as raw frequencies and relative percentages. Differences in distributions for categorical data between two or more groups were assessed using the Chi-square test, or the Fisher’s exact test in case of insufficient sample size.

Time-to-event data were analysed using the Kaplan-Meier estimator to deal with interval-censored data. Results were presented as cumulative proportion curves.

Multivariate logistic regression was used to assess the association of multiple factors with the occurrence of joint EIM. First, all factors with univariate p-value < 0.200 were included into the multivariate model. Non-significant factors (p>0.05) were then excluded from the model one-by-one, until all remaining factors were significant. In a last step, all factors that were left aside were once more inserted one-by-one into the model and kept if proven significant, while checking for model consistency at each step of the procedure.

### Ethical considerations

The IBD cohort study has been approved by the respective ethics committees in Switzerland (Ethics Committee of the Canton Zürich: EK-1316). All patients signed an informed consent, confirmed their participation in the cohort study at the time of enrollment and gave informed consent for data collection and analysis for research purposes. For inclusion of minors, obtained consent had been provided from parents or guardians. The current sub-study has been evaluated and approved by the scientific board of SIBDCS.

## Results

### Epidemiology of arthritis/arthralgia in CD patients

From the 1860 CD patients included in the SIBDC, 907 respectively 48.8% suffered from arthritis/arthralgia during their disease course. Female CD patients were found to be significantly more often affected with joint EIM than men. Age at diagnosis of CD patients with joint manifestations was significantly higher than the age at diagnosis of CD patients without joint EIM (median 27 vs. 25 years). Furthermore, patients with arthritis/arthralgia included in the SIBDCS suffered from a significantly longer disease duration of 15 years than patients without arthritis/arthralgia with only 11 years (Table 1). BMI and smoking status showed no association with the presence of arthritis/arthralgia.

**Table 1:** Relation between epidemiological factors (gender, age, disease duration) and joint manifestations in CD patients.

	No Joint EIM	Joint EIM	All CD patients	pvalue
**Number of patients**	953 (51.2)	907 (48.8)	1860 (100.0)	
**Gender**				
**Male**	520 (54.6)	366 (40.4)	886 (47.6)	
**Female**	433 (45.4)	541 (59.6)	974 (52.4)	<0.001
**Age at diagnosis [y] (median,IQR, range)**	25, 20–35, 4–81	27, 20–38, 1–81	26, 20–37, 1–81	0.004
**Age at latest follow-up [y] (median, IQR, range)**	40, 31–53, 17–89	48, 36–60, 16–94	44, 33–57, 16–94	<0.001
**Disease duration [y]** **(median, IQR, range)**	11, 6–20, 0–53	15, 9–24, 0–57	13, 7–22, 0–57	<0.001
**Smoking status at diagnosis**				
**Non-smoker**	488 (51.2)	426 (47.0)	914 (49.1)	
**Smoker**	420 (44.1)	445 (49.1)	865 (46.5)	
**Unknown**	45 (4.7)	36 (4.0)	81 (4.4)	0.091
**Smoking status at latest follow up**				
**Non-Smoker**	662 (69.5)	601 (66.3)	1263 (67.9)	
**Smoker**	282 (29.6)	302 (33.3)	584 (31.4)	
**Unknown**	9 (0.9)	4 (0.4)	13 (0.7)	0.110

### Disease characteristics in CD patients

We did not find any association of arthritis/arthralgia and initial disease location. However, ileocolic inflammation (L3) was the most common disease location for both patient groups at the beginning of the disease. CDAI (Crohn’s disease activity index) at enrollment (median 51 vs. 21) as well as at follow-up (33 vs. 13) was significantly higher in patients with arthritis/arthralgia (Table 2).

**Table 2:** Disease characteristics (location and disease activity), disease complications (fistula, abscess, stenosis), CD-related surgery and other EIMs in relation to joint EIM in CD patients.

	No Joint EIM	Joint EIM	All CD patients	pvalue
**Number of patients**	953 (51.2)	907 (48.8)	1860 (100.0)	
**Initial disease location**				
**L1**	244 (25.6)	201 (22.2)	445 (23.9)	
**L2**	184 (19.3)	182 (20.1)	366 (19.7)	
**L3**	414 (43.4)	415 (45.8)	829 (44.6)	
**L4 only**	7 (0.7)	7 (0.8)	14 (0.8)	
**Unclear/Unknown**	104 (10.9)	102 (11.3)	206 (11.1)	0.549
**Last disease location**				
**L1**	284 (29.8)	237 (26.1)	521 (28.0)	
**L2**	266 (27.9)	290 (32.0)	556 (29.9)	
**L3**	267 (28.0)	246 (27.1)	513 (27.6)	
**L4 only**	13 (1.4)	34 (3.4)	47 (2.5)	
**Unclear/Unknown**	123 (12.9)	100 (11.0)	223 (12.0)	0.002
**CDAI at enrolment (median, IQR, range)**	21, 6–53,0–280	51, 26–93, 0–450	34, 12–76, 0–450	<0.001
**CDAI at latest follow-up (median, IQR, range)**	13, 6–26, 0–218	33, 11–65, 0–395	21, 6–51, 0–395	<0.001
**CD-related complications**				
**Perianal Fistula**	235 (24.7)	246 (27.1)	481 (25.9)	0.225
**Other Fistula**	145 (12.2)	158 (17.4)	303 (16.3)	0.198
**Any Fistula**	327 (34.3)	323 (35.6)	650 (34.9)	0.557
**Abscess**	223 (23.4)	238 (26.2)	461 (24.8)	0.156
**Stenosis**	3876 (40.5)	418 (46.1)	804 (43.2)	0.015
**CD-related surgery**				
**Intestinal resection**	366 (38.4)	405 (44.7)	771 (41.5)	0.006
**Fistula/Abscess surgery**	233 (24.5)	235 (25.9)	468 (25.2)	0.468
**Any Surgery**	469 (49.2)	486 (53.6)	955 (51.3)	0.059
**Anemia**				
**During SIBDCS follow-up**	242 (26.0)	315 (35.1)	557 (30.5)	<0.001
**At latest follow-up**	85 (11.3)	98 (13.7)	183 (12.5)	0.170
**Vit. B12 level at latest follow-up [pmo]/l)** **(median, IQR, range)**	231, 171–309, 37–2435	250, 186–342, 28–1500	242, 178–325, 28–2435	0.003
**Ever received Vit. B12 Suppl.**	334 (45.6)	459 (57.0)	793 (51.6)	<0.001
**Extraintestinal manifestations**				
**Uveitis/Iritis**	41 (4.3)	166 (18.3)	207 (11.1)	<0.001
**Pyoderma Gangrenosum**	10 (1.0)	19 (2.1)	29 (1.6)	0.069
**Erythema Nodosum**	35 (3.7)	108 (11.9)	143 (7.7)	<0.001
**Aphtous/oral ulcers**	55 (5.8)	187 (20.6)	242 (13.0)	<0.001
**Ankylosing spondilitis**	18 (1.9)	110 (12.1)	128 (6.9)	<0.001
**PSC**	7 (0.7)	5 (0.6)	12 (0.6)	0.775
**any of the above**	143 (15.09	414 (45.6)	557 (29.9)	<0.001

### Disease complications and other EIM in CD patients

CD-related complications such as perianal fistula, other fistula, any fistula and abscesses revealed not to be associated to joint EIM in CD patients. However, CD patients with arthritis/arthralgia suffered significantly more often from a structuring disease course and a subsequent stenosis. Subsequently, patients suffering from arthritis/arthralgia underwent intestinal resection more often than patients without arthritis/arthralgia. Yet, there was no difference detected between the two groups for specific fistula/abscess surgery. This implies that patients with arthritis/arthralgia might preferably present with a B2 rather than a B3 disease behavior.

To assess an association of malabsorption with the onset of arthritis/arthralgia in CD patients, we next analyzed, whether anemia and low vitamin B12 serum levels were more frequent in patients with or without arthritis/arthralgia. During SIBDCS follow-up, anemia was more often observed in patients with arthritis/arthralgia as compared to patients without. Vitamin B12 levels at latest follow-up did not indicate a deficit in both patient groups (with and without joint EIM). However patients with arthritis/arthralgia had higher levels of serum vitamin B12 (250 vs. 231 pmol/l) overall. Furthermore, patients with arthritis/arthralgia received more often a vitamin B12 supplementation at any time during the observation period than patients with no arthritis/arthralgia, likely because of the more severe disease and therefore higher risk for malabsorption.

We found other EIM to be significantly associated with the existence of arthritis/arthralgia in CD patients. Uveitis/Iritis (18.3% vs. 4.3%), erythema nodosum (11.9% vs. 3.7%) and aphtous/oral ulcers (20.6% vs. 5.8%) were significantly more frequent in CD patients with existing arthritis/arthralgia. In contrast, pyoderma gangrenosum and PSC did not show a significant difference between both of the groups (Table 2).

### Treatment history in CD patients

We further analyzed the treatment history of CD patients with and without joint EIM. Patients with arthritis/arthralgia received significantly more often 5-ASA, antibiotics, steroids, immunomodulators and anti-TNF (Table 3). To further validate our findings, we next performed a multivariate logistic regression analysis for possible factors being associated with the presence of arthritis/arthralgia in CD patients. Here, we detected that female gender, older age at diagnosis, a longer disease duration, IBD-related surgery not due to fistula or abscesses, 5-ASA treatment, treatment with steroids, treatment with anti-TNF as well as the presence of other EIM were independently associated with the occurrence of arthritis/arthralgia in the CD patients of the SIBDCS patient collective (Table 4).

**Table 3:** Relation between treatment history and joint manifestations in CD patients.

	No Joint EIM	Joint EIM	All CD patients	pvalue
**Number of patients**	953 (51.2)	907 (48.8)	1860 (100.0)	
**Treatment history**				
5-ASA	489 (51.3)	580 (63.9)	1069 (57.5)	<0.001
Antibiotics	143 (15.0)	197 (21.7)	340 (18.3)	<0.001
Steroids	781 (82.0)	821 (90.5)	1602 (86.1)	<0.001
Immunomodulators	750 (78.7)	764 (84.2)	1514 (81.4)	0.002
Anti-TNF	543 (57.0)	637 (70.2)	1180 (63.4)	<0.001
Calcineurin inhibitors	13 (1.4)	23 (2.5)	36 (1.9)	0.067

**Table 4:** Multivariate logistic regression results for CD patients.

Outcome: Joint EIM	OR (95% CI)	pvalue
**Gender**		
Male	1 (ref)	
Female	1.711 (1.314–2.229)	<0.001
**Age at diagnosis [per year]**	1.030 (1.020–1.040)	<0.001
**Disease duration [per year]**	1.048 (1.032–1.063)	<0.001
**CDAI at latest follow-up** **[per CDAI point]**	1.010 (1.006–1.013)	<0.001
**IB-related surgery**		
Non (ref)	1 (ref)	
Yes	0.743 (0.556–0.993)	0.045
**5-ASA treatment**		
Never (ref)	1 (ref)	
Yes	1.340 (1.017–1.766)	0.038
**Steroids treatment**		
Never (ref)	1 (ref)	
Yes	1.837 (1.163–2.900)	0.009
**Anti-TNF treatment**		
Never (ref)	1 (ref)	
Yes	1.693 (1.265–2.265)	<0.001
**Other EIM**		
No (ref)	1 (ref)	
Yes	4.710 (3.494–6.349)	<0.001

### Epidemiology of joint manifestations in UC/IBDU patients

Out of 1438 UC/IBDU patients, 34.6% (498 patients; 231 male and 267 female patients, respectively) suffered from arthritis/arthralgia. Similar to CD patients, age at diagnosis of UC/IBDU patients with arthritis/arthralgia was slightly higher as compared to UC/IBDU patients without arthritis/arthralgia (31 vs. 30 years). Median disease duration for patients with arthritis/arthralgia was 13 years, while it was only 11 years in patients without arthritis/arthralgia. In contrast to our findings for CD patients, smoking at diagnosis of UC/IBDU patients was significantly related to the presence of arthritis/arthralgia (Table 5).

**Table 5:** Relation between epidemiological factors and the presence of joint EIM in UC/IBDU patients.

	No Joint EIM	Joint EIM	All UC/IBDU pat.	pvalue
**Number of patients**	940 (65.4)	498 (34.6)	1438 (100.0)	
**Gender**				
Male	542 (27.7)	231 (46.4)	773 (53.8)	
Female	398 (42.3)	267 (53.6)	665 (46.2)	<0.001
**Age at diagnosis [y] (median,IQR, range)**	30, 23–40, 3–83	31, 25–42, 10–80	31, 23–41, 3–83	0.032
**Age at latest follow-up [y] (median, IQR, range)**	44, 35–55, 17–88	49, 39–59, 22–89	46, 36–57, 17–89	<0.001
**Disease duration [y]** **(median, IQR, range)**	11, 6–17, 0–59	13, 9–21, 0–52	12, 7–19, 0–59	<0.001
**Smoking status at diagnosis**				
Non-smoker	723 (76.9)	359 (72.1)	1082 (75.2)	
Smoker	176 (18.7)	126 (25.3)	302 (21.0)	
Unknown	41 (4.4)	13 (2.6)	54 (3.8)	0.006
**Smoking status at latest follow up**				
Non-Smoker	799 (85.0)	420 (84.3)	1219 (84.8)	
Smoker	126 (13.4)	73 (14.7)	199 (13.8)	
Unknown	15 (1.6)	5 (1.0)	20 (1.4)	0.547

### Disease characteristics in UC/IBDU patients

We detected no difference between initial and last disease location and appearance of arthritis/arthralgia in UC/IBDU patients. However, disease activity at enrollment and at latest follow-up was significantly higher for UC/IBDU patients with arthritis/arthralgia compared to patients without arthritis/arthralgia. Median MTWAI at enrollment for patients with arthritis/arthralgia was 3 and the median for patients without arthritis/arthralgia was 2 (Median MTWAI at latest follow-up: 2 vs. 1; Table 6).

**Table 6:** Disease characteristics (disease location, disease activity), disease complications and other EIMs in relation to joint EIM in UC/IBDU patients.

	No Joint EIM	Joint EIM	All UC/IBDU pat.	pvalue
**Number of patients**	940 (65.4)	498 (34.6)	1438 (100.0)	
**Initial disease location**				
**Pancolitis**	348 (37.0)	191 (38.4)	539 (37.5)	
**Left-sided colitis**	309 (32.9)	153 (30.7)	462 (32.1)	
**Proctitis**	190 (20.2)	93 (18.7)	283 (19.7)	
**Unclear/Unknown**	93 (9.9)	61 (12.3)	154 (10.7)	0.440
**Last disease location**				
**Pancolitis**	328 (34.9)	168 (33.7)	496 (34.5)	
**Left-sided colitis**	335 (35.6)	187 (37.6)	522 (36.3)	
**Proctitis**	171 (18.2)	84 (16.9)	255 (17.7)	
**Unclear/Unknown**	106 (11.3)	59 (11.9)	165 (11.5)	0.833
**MTWAI at enrollment** **(median, IQR, range)**	2, 1–5, 0–19	3, 1–6, 0–18	2, 1–5, 0–19	<0.001
**MTWAI at latest follow-up** **(median, IQR, range)**	1, 0–3, 0–18	2, 1–4, 0–18	2, 0–4, 0–18	<0.001
**IBD-related surgery**	82 (8.7)	78 (15.7)	160 (11.1)	<0.001
**Anemia**				
**During SIBDCS follow-up**	252 (29.3)	165 (34.2)	417 (31.1)	0.067
**At latest follow-up**	82 (13.1)	52 (14.2)	134 (13.5)	0.622
**Vit. B12 level at latest follow-up [pmo]/l)** **(median, IQR, range)**	299, 209–371, 54–1696	270, 202–363, 41–1476	279, 207–369, 41–1696	0.237
**Ever received Vit. B12 Suppl.**	139 (20.7)	116 (26.7)	255 (23.1)	0.021
**Extraintestinal manifestations**				
**Uveitis/Iritis**	28 (3.0)	52 (10.4)	80 (5.6)	<0.001
**Pyoderma Gangrenosum**	10 (1.1)	15 (3.0)	25 (1.7)	0.007
**Erythema Nodosum**	14 (1.5)	37 (7.4)	51 (3.5)	<0.001
**Aphtous/oral ulcers**	22 (2.3)	57 (11.4)	79 (5.5)	<0.001
**Ankylosing spondilitis**	14 (1.5)	30 (6.0)	44 (3.1)	<0.001
**PSC**	40 (4.3)	21 (4.2)	61 (4.2)	0.973
**any of the above**	112 (11.9)	157 (31.5)	269 (18.7)	<0.001

### Disease complications and other EIM in UC/IBDU patients

Significantly more patients with arthritis/arthralgia ever had an UC/IBDU-related surgery, likely colectomy, compared to patients without arthritis/arthralgia. Additionally, patients with arthritis/arthralgia received more often vitamin B12 supplementation than patients without arthritis/arthralgia, although there was no difference in vitamin B12 levels at latest follow-up or in the presence of anaemia between the two groups. In accordance with previous data and our findings in CD patients, UC/IBDU patients with arthritis/arthralgia more frequently suffered from other EIM. 10.4% vs. 3.0% presented with uveitis/iritis, 3.0% vs. 1.1% with pyoderma gangrenosum, 7.4% vs. 1.5% with erythema nodosum, 11.4% vs. 2.3% with aphtous/oral ulcers and 6.0% vs. 1.5% with ankylosing spondylitis. No difference between the groups was detected for the presence of PSC (Table 6).

### Treatment history and joint manifestations in UC/IBDU patients

Antibiotics, steroids, immunomodulators, anti-TNF and calcineurin inhibitors were significantly more often used in patients with arthritis/arthralgia. The use of 5-ASA was similar between the groups (Table 7). To further validate our findings, we next performed a multivariate logistic regression analysis for possible factors being associated with the presence of arthritis/arthralgia in UC/IBDU patients. Here, similar to CD patients, we identified female gender, older age at diagnosis, longer disease duration, IBD-related surgery, treatment with anti-TNF as well as the presence of other EIM to be independently associated with the presence of arthritis/arthralgia in the UC/IBDU. Additionally, also smoking was associated with an increased risk for arthritis/arthralgia in UC/IBDU patients (Table 8).

**Table 7:** Treatment history and appearance of joint manifestations in UC/IBDU patients.

	No Joint EIM	Joint EIM	All UC/IBDU	pvalue
**Number of patients**	940 (65.4)	498 (34.6)	1438 (100.0)	
**Treatment history**				
**5-ASA**	885 (94.1)	478 (96.0)	1363 (94.8)	0.136
**Antibiotics**	86 (9.1)	72 (14.5)	158 (11.0)	0.002
**Steroids**	103 (74.8)	439 (88.2)	1142 (79.4)	<0.001
**Immunomodulators**	529 (56.3)	353 (70.9)	882 (61.3)	<0.001
**Anti-TNF**	286 (30.4)	215 (43.2)	501 (34.8)	<0.001
**Calcineurin inhibitors**	79 (8.4)	60 (12.0)	139 (9.7)	0.026

**Table 8:** Multivariate logistic regression results for UC patients.

Outcome: Joint EIM	OR (95% CI)	pvalue
**Gender**		
**Male**	1 (ref)	
**Female**	1.792 (1.346–2.387)	<0.001
**Age at diagnosis [per year]**	1.027 (1.016–1.039)	<0.001
**Disease duration [per year]**	1.040 (1.024–1.057)	<0.001
**Smoking status at diagnosis**		
**Non-smoker (ref)**	1 (ref)	
**Smoker**	1.705 (1.214–2.395)	0.002
**IBD-relates surgery**		
**None (ref)**	1 (ref)	
**Yes**	1.542 (1.040–2.285)	0.031
**Anti-TNF treatment**		
**Never (ref)**	1 (ref)	
**Yes**	1.644 (1.231–2.196)	0.001
**Other EIM**		
**No (ref)**	1 (ref)	
**Yes**	3.132 (2.239–4.381)	<0.001

### Chronology of arthritis/arthralgia in CD and UC/IBDU patients

**Fig 1:**
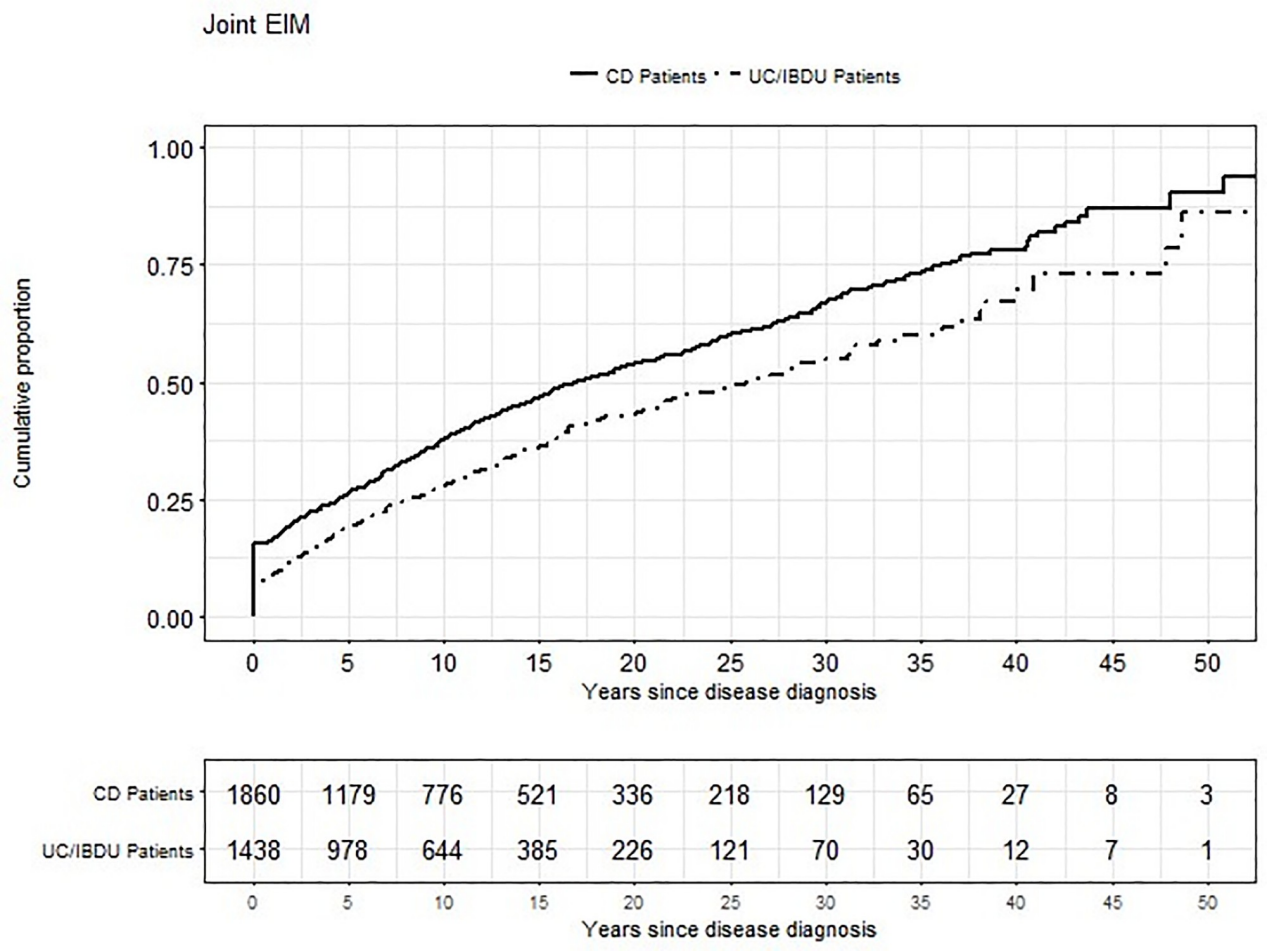
Cumulative proportion of arthritis / arthralgia.

Finally, we studied whether arthritis/arthralgia appear as first or subsequent EIM in IBD patients. We found that 50 years after IBD diagnosis almost every patient suffered from arthritis/arthralgia (Fig 1). Arthritis/arthralgia seem to present rather as first EIM than as subsequent EIM–this applies for all IBD patients together (83.3% reported arthritis/arthralgia as first EIM vs. 16.7% who reported arthritis/arthralgia as subsequent EIM, p = 0.449). 80.4% of all IBD patients reported arthritis/arthralgia occurrence after the diagnosis of IBD.

### Ankylosing spondilitis/Sacroliitis in IBD patients

We next performed a further analysis of our patient collective of 3298 patients and analyzed the associations of the clinical presentation of the patients and the presence or absence of sacroiliitis/ankylosing spondylitis. We found that only 155 (8.3%) of the 1860 CD patients were suffering from specifically sacroiliitis/ankylosing spondylitis compared to 907 (48.8%) of our CD patients suffering from arthralgia/arthritis. CD patients with sacroiliitis/ankylosing spondylitis were in general older at diagnosis than patients without sacroiliitis/ankylosing spondylitis during disease course (29 vs 26 years), but featured a longer disease duration (15 vs 13 years). While they did not reveal more CD-related complications, such as fistulas, abscesses or stenosis, or surgeries, they more frequently received vitamin B12 supplementation, mesalazin (5-ASA) and anti-TNF antibodies when compared to CD patients without sacroiliitis/ankylosing spondylitis. Notably CD patients with sacroiliitis/ankylosing spondylitis suffered significantly more often from other EIM, such as uveitis, iritis, erythema nodosum or aphthous/oral ulcers (Table 9).

**Table 9:** Clinical associations of CD patients with ankylosing spondilitis / sacroliitis.

	No Anky spond./Sacro	Anky spond./Sacro	All CD patients	pvalue
**Number of patients**	1705 (91.7)	155 (8.3)	1860 (100.0)	
**Gender**				
**Male**	818 (48.0)	68 (43.9)	886 (47.6)	
**Female**	887 (52.0)	87 (56.1)	974 (52.4)	0.327
**Age at diagnosis [y] (median,IQR, range)**	26, 20–36,1–81	29, 22–41, 11–71	26, 20–37, 1–81	0.010
**Age at latest follow-up [y] (median, IQR, range)**	44, 33–56, 16–94	49, 37–60, 22–84	44, 33–57, 16–94	0.003
**Disease duration [y]** **(median, IQR, range)**	13, 7–22, 0–57	15, 9–22, 0–46	13, 7–22, 0–57	0.049
**BMI at latest follow-up [kg/m2]** **(median, IQR, range)**	23, 20–26, 14–43	23, 22–25, 19–29	23, 20–26, 14–43	0.882
**Initial disease location**				
**L1**	408 (23.9)	37 (23.9)	445 (23.9)	
**L2**	329 (19.3)	37 (23.9)	366 (19.7)	
**L3**	763 (44.8)	66 (42.3)	829 (44.6)	
**L4 only**	13 (0.8)	1 (0.7)	14 (0.8)	
**Unclear/Unknown**	192 (11.3)	14 (9.0)	206 (11.1)	0.661
**Last disease location**				
**L1**	469 (27.5)	52 (33.6)	521 (28.0)	
**L2**	522 (30.6)	34 (21.9)	556 (29.9)	
**L3**	469 (27.5)	44 (28.4)	513 (27.6)	
**L4 only**	41 (2.4)	6 (3.9)	47 (2.5)	
**Unclear/Unknown**	204 (12.0)	19 (12.3)	223 (12.0)	0.153
**CDAI at enrollment (median, IQR, range)**	33, 11–74, 0–435	53, 26–91, 0–450	34, 12–76, 0–450	<0.001
**CDAI at latest follow-up (median, IQR, range)**	20, 6–48, 0–395	40, 20–83, 0–345	21, 6–51, 0–395	<0.001
**Smoking status at diagnosis**				
**Non-smoker**	845 (49.6)	69 (44.5)	914 (49.1)	
**Smoker**	781 (45.8)	84 (54.2)	865 (46.5)	
**Unknown**	79 (4.6)	2 (1.3)	81 (4.4)	0.034
**Smoking status at latest follow-up**				
**Non-smoker**	1158 (67.9)	105 (67.7)	1263 (67.9)	
**Smoker**	534 (31.3)	50 (32.3)	584 (31.4)	
**Unknown**	13 (0.8)	0 (0.0)	13 (0.7)	0.773
**CD-related complications**				
**Perianal Fistula**	445 (26.1)	36 (23.2)	481 (25.9)	0.434
**Other Fistula**	283 (16.6)	20 (12.9)	303 (16.3)	0.233
**Any Fistula**	601 (35.2)	49 (31.6)	650 (34.9)	0.363
**Abscess**	419 (24.6)	42 (27.1)	461 (24.8)	0.486
**Stenosis**	733 (43.0)	71 (45.8)	804 (43.2)	0.498
**CD-related surgery**				
**Intestinal resection**	701 (41.1)	0 (45.2)	771 (41.5)	0.327
**Fistula/Abscess surgery**	431 (25.3)	37 (23.9)	468 (25.2)	0.699
**Any Surgery**	874 (51.3)	81 (52.3)	955 (51.3)	0.812
**Anemia**				
**During SIBDCS follow-up**	500 (29.9)	57 (37.3)	557 (30.5)	0.057
**At latest follow-up**	165 (12.3)	18 (14.4)	183 (12.5)	0.492
**Vit. B12 level at latest follow-up [pmo]/l)** **(median, IQR, range)**	243, 178–324, 28–2435	242, 191–325, 74–1258	242, 178–325, 28–2435	0.802
**Ever received Vit. B12 Suppl.**	709 (50.6)	84 (61.8)	793 (51.6)	0.013
**Treatment history**				
**5-ASA**	965 (56.6)	104 (67.1)	1069 (57.5)	0.011
**Antibiotics**	311 (18.2)	29 (18.7)	340 (18.3)	0.885
**Steroids**	1468 (86.1)	134 (86.5)	1602 (86.1)	0.903
**Immunomodulators**	1386 (81.3)	128 (82.6)	1514 (81.4)	0.963
**Anti-TNF**	1059 (62.1)	121 (78.1)	1180 (63.4)	<0.001
**Calcineurin inhibitors**	31 (1.8)	5 (3.2)	36 (1.9)	0.218
**Extraintestinal manifestations**				
**Arthritis/Arthalgia**	770 (45.2)	137 (88.4)	907 (48.8)	<0.001
**Uveitis/Iritis**	167 (9.8)	40 (25.8)	29 (1.6)	<0.001
**Pyoderma Gangrenosum**	26 (1.5)	3 (1.9)	143 (7.7)	0.729
**Erythema Nodosum**	123 (7.2)	20 (12.9)	242 (13.0)	0.011
**Aphtous/oral ulcers**	203 (11.9)	39 (25.2)	128 (6.9)	<0.001
**PSC**	10 (0.6)	2 (1.3)	12 (0.6)	0.264
**any of the above**	895 (52.5)	140 (90.3)	1035 (55.6)	<0.001
**Family history of IBD**				
**None**	1297 (76.1)	116 (74.8)	1413 (76.0)	
**Yes**	222 (13.0)	28 (18.1)	250 (13.4)	
**Unknown**	186 (10.9)	11 (7.1)	197 (10.6)	0.097

Only 63 (4.4%) of the 1438 UC/IBDU patients suffered from specifically sacroiliitis/ankylosing spondylitis compared to 498 (34.6%) of our UC/IBDU patients suffering from arthralgia/arthritis. UC/IBDU patients with sacroiliitis/ankylosing spondylitis received more frequently vitamin B12 supplementation and anti-TNF antibodies when compared to UC/IBDU patients without sacroiliitis/ankylosing spondylitis. Similar to CD patients, also UC/IBDU patients with sacroiliitis/ankylosing spondylitis suffered significantly more often from other EIM, such as uveitis, iritis or aphthous/oral ulcers (Table 10).

**Table 10:** Clinical associations of UC/IBDU patients with ankylosing spondilitis / sacroliitis.

	No Anky spond./Sacro	Anky spond./Sacro	All UC/IBDU pat.	pvalue
**Number of patients**	1375 (95.6)	63 (4.4)	1438 (100.0)	
**Gender**				
**Male**	741 (53.9)	32 (50.8)	773 (53.8)	
**Female**	634 (46.1)	31 (49.2)	665 (46.2)	0.630
**Age at diagnosis [y] (median,IQR, range)**	31, 32–40, 3–83	31, 23–42, 12–66	321, 23–41, 3–83	0.644
**Age at latest follow-up [y] (median, IQR, range)**	46, 36–57, 17–89	47, 39–59, 22–79	46, 36–57, 17–89	0.256
**Disease duration [y]** **(median, IQR, range)**	12, 7–19, 0–59	13, 9–18, 2–49	12, 7–19, 0–59	0.071
**BMI at latest follow-up [kg/m2]** **(median, IQR, range)**	24, 22–27, 18–38	22, 19–27, 17–30	24, 22–27, 17–38	0.391
**Initial disease location**				
**Pancolitis**	517 (37.6)	22 (34.9)	539 (37.5)	
**Left-sided colitis**	435 (31.6)	27 (42.9)	462 (32.1)	
**Proctitis**	272 (19.8)	11 (17.5)	283 (19.7)	
**Unclear/Unknown**	151 (11.0)	3 (4.8)	154 (10.7)	0.205
**Last disease location**				
**Pancolitis**	476 (34.6)	20 (31.8)	496 (34.5)	
**Left-sided colitis**	496 (36.1)	26 (41.3)	522 (36.3)	
**Proctitis**	243 (17.7)	12 (19.1)	255 (17.7)	
**Unclear/Unknown**	160 (11.6)	5 (7.9)	165 (11.5)	0.746
**MTWAI at enrollment (median, IQR, range)**	2, 1–5, 0–19	4, 1–7, 0–15	2, 1–5, 0–19	0.010
**MTWAI at latest follow-up (median, IQR, range)**	2, 0–3, 0–18	3, 1–4, 0–18	2, 0–4, 0–18	0.059
**Smoking status at diagnosis**				
**Non-smoker**	1034 (75.2)	48 (76.2)	1082 (75.2)	
**Smoker**	288 (21.0)	14 (22.2)	302 (21.0)	
**Unknown**	53 (3.8)	1 (1.6)	54 (3.8)	0.790
**Smoking status at latest follow-up**				
**Non-smoker**	1168 (85.0)	51 (81.0)	1219 (84.4)	
**Smoker**	187 (13.6)	12 (19.0)	199 (13.8)	
**Unknown**	20 (1.4)	0 (0.0)	20 (1.4)	0.422
**IBD-related surgery**	149 (10.8)	11 (17.5)	160 (11.1)	0.102
**Anemia**				
**During SIBDCS follow-up**	401 (31.3)	16 (26.7)	417 (31.1)	0.451
**At latest follow-up**	128 (13.5)	6 (13.6)	134 (13.5)	1.000
**Vit. B12 level at latest follow-up [pmo]/l)** **(median, IQR, range)**	285, 208–371, 41–1696	242, 201–324, 110–1476	279, 207–369, 41–1696	0.261
**Ever received Vit. B12 Suppl.**	235 (22.4)	20 (35.1)	255 (23.1)	0.027
**Treatment history**				
**5-ASA**	1302 (94.7)	61 (96.8)	1363 (94.8)	0.456
**Antibiotics**	150 (10.9)	8 (12.7)	158 (11.0)	0.657
**Steroids**	1088 (79.1)	54 (85.7)	1142 (79.4)	0.206
**Immunomodulators**	836 (60.8)	46 (73.0)	882 (61.3)	0.052
**Anti-TNF**	468 (34.0)	33 (52.4)	501 (34.8)	0.003
**Calcineurin inhibitors**	131 (9.5)	8 (12.7)	139 (9.7)	0.405
**Extraintestinal manifestations**				
**Arthritis/Arthalgia**	453 (32.96)	45 (71.4)	498 (38.5)	<0.001
**Uveitis/Iritis**	64 (4.7)	16 (25.4)	25 (1.7)	<0.001
**Pyoderma Gangrenosum**	21 (1.5)	4 (6.3)	51 (3.5)	0.021
**Erythema Nodosum**	47 (3.4)	6 (6.4)	79 (5.5)	0.279
**Aphtous/oral ulcers**	70 (5.1)	9 (14.3)	44 (3.1)	0.002
**PSC**	58 (4.2)	3 (4.8)	61 (4.2)	0.747
**any of the above**	550 (40.0)	52 (82.5)	602 (41.9)	<0.001
**Family history of IBD**				
**None**	1087 (79.1)	51 (81.0)	1138 (79.1)	
**Yes**	141 (10.2)	0 (14.3)	150 (10.4)	
**Unknown**	147 (10.7)	3 (4.7)	150 (10.4)	0.210

**Fig 2:**
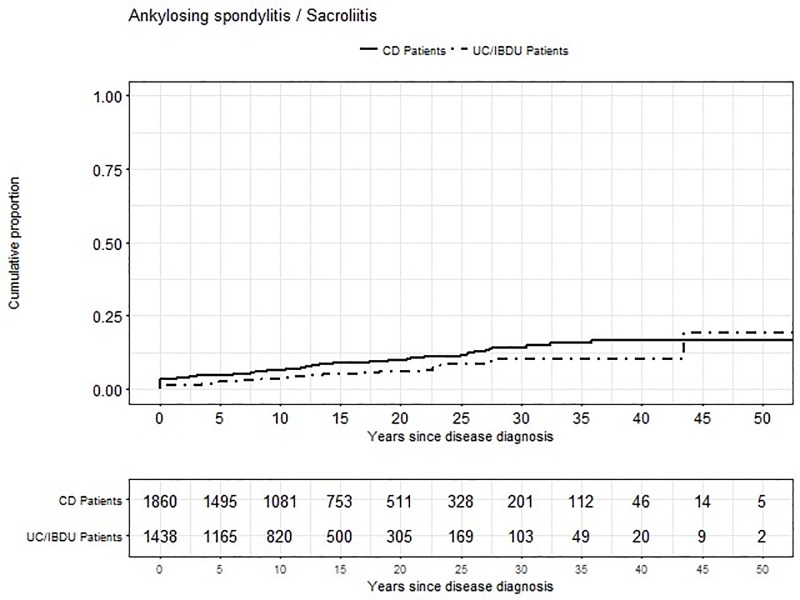
Cumulative proportion of ankylosing spondylitis / sacroliitis.

Finally, we found that 50 years after IBD diagnosis about 20% of patients suffered from sacroiliitis/ankylosing spondylitis (Fig 2). Similar to arthritis/arthralgia, also sacroiliitis/ankylosing spondylitis is reported rather as first EIM than as subsequent EIM–this applies for all IBD patients together (76.1% reported sacroiliitis/ankylosing spondylitis as first EIM vs. 23.9% who reported sacroiliitis/ankylosing spondylitis as subsequent EIM, p = 0.702). 39.1% reported sacroiliitis/ankylosing spondylitis already before IBD diagnosis.

## Discussion

In this study, we aimed to find epidemiologic and clinical risk factors for the occurrence of arthritis/arthralgia in patients suffering from IBD. We found that females are more likely to develop arthritis/arthralgia regardless whether they suffer from CD or UC. This observation is in good accordance with previous data from the SIBDCS, where 50% of the female CD patients, but only 34% of male CD patients suffered from any EIM [1, 18]. Male IBD patients however, more commonly present with axial arthropathies than female patients [3, 19]. Our study has now analyzed the presence of EIM in more detail and focused on arthritis/arthralgia and, in a second step, on the subgroup of patients with sacroiliitis/ankylosing spondylitis.

We found that arthritis/arthralgia are more common in CD and UC/IBDU patients with a higher disease activity. This might be plausible, since patients with more severe intestinal inflammation are also likely to suffer more frequently from pain overall and therefore also from joint pain. This finding has already been described for UC patients in a study from 1990, where 70% of patients with extracolonic manifestations suffered from highly active UC [20]. We also found evidence that the disease duration is associated with the presence of arthritis/arthralgia as well. After 5 years about 25% of CD patients suffer from arthritis/arthralgia and after 15 years, already about 50% of CD patients suffer from such manifestations. A longer disease duration and a higher disease activity are plausible markers for a severe disease course that is also associated with intestinal surgery due to IBD. *Cleynen et al*. have nicely shown that the time between diagnosis of IBD and occurrence of complications or surgery is dependent on disease location, particularly colonic inflammation, in CD patients [21], whereas for UC patients, disease progression is mainly dependent on the extent of the inflammation [21]. We found that a longer disease duration as well as higher disease activity are associated with the presence of arthritis/arthralgia in IBD patients. A recent study further found that in pediatric patients exhibiting a respectively young age at diagnosis what also hints towards a more severe disease course, is associated with onset of EIM later on [22].

CD and UC/IBDU patients with arthritis/arthralgia were significantly more often treated with anti-TNF when compared to patients without arthritis/arthralgia. This might on the one hand reflect that patients with arthritis/arthralgia are suffering from a more severe disease and therefore need more frequently anti-TNF treatment. On the other hand, anti-TNF antibodies are commonly used for treating arthritis and arthritis/arthralgia in IBD patients. Those observations have also been confirmed in a pediatric cohort of IBD patients [23]. Of interest, pediatric CD patients seem to suffer clearly more frequently from EIM and in particular joint EIM than elderly-onset IBD patients [24].

Limitations of our analysis are certainly on the one hand that we are lacking the info which joint EIM occurred before diagnosis of IBD and on the other hand, that joint disease associated with IBD was not confirmed by a rheumatologist. This suggests that an accurate diagnosis of the underlying diagnosis of arthritis/arthralgia might not be available in any patient. However, to improve the quality of our data, we also analyzed the clinical associations of patients suffering from sacroiliitis/ankylosing spondylitis as a clear and accurate diagnosis. Interestingly, though for this patient group, the number of affected patients was clearly less, we found comparable disease characteristics.

With respect to our data, however, a recent publication by Ossum et al. it noteworthy. Here, using the inception cohort from the IBSEN study, the authors followed IBD patients prospectively for 20 years with respect to the prevalence of IBD-related peripheral arthritis and peripheral spondyloarthritis. The authors found that prevalence of developing disease- (IBD-) related peripheral arthritis or peripheral spondyloarthritis during disease course was 17.2% or 27.9%, respectively. Those data are somehow in the range that we observe in our study using the patient collective of the SIBDCS. In line with our data, also in the IBSEN study, more women than men suffered from IBD-related peripheral arthritis and peripheral spondyloarthritis [25]. Those data are also in line with data from India. In this study, a trained rheumatologist confirmed the diagnosis and prevalence for joint EIM was found to be 23% for peripheral arthritis and 18% for ankylosing spondylitis during disease course [26]. In contrast, a previous study from Europe reported a clearly lower prevalence for unspecified arthritis as EIM in European IBD patients [27].

Of note, we detected no increased risk to develop joint EIM in smoking CD patients compared to non-smoking CD patients and observed that smoking UC patients are actually protected from the onset of joint EIM when compared to non-smoking UC patients. This is in contrast to data from Severs et al. who found by analyzing data from three large cohorts that joint manifestations might be more prevalent in IBD patients that smoke than in those who not smoke. Interestingly, they found this association for both, CD and UC patients [28]. At least for UC patients, this finding is somehow surprising, since smoking is generally considered to protect from UC and to be associated with a less severe disease course in UC patients [29].

In conclusion, we found a more severe disease course to be associated with arthritis/arthralgia, but also with sacroiliitis/ankylosing spondylitis in IBD patients. In particular, anti-TNF treatment, onset of further EIM and IBD-related surgery which are all indicative of a severe course of disease, are risk factors for the presence of arthritis/arthralgia, but also with sacroiliitis/ankylosing spondylitis in IBD patients.
